# Cross-sectional analysis of health-related quality of life and elements of yoga practice

**DOI:** 10.1186/s12906-017-1599-1

**Published:** 2017-01-31

**Authors:** Gurjeet S. Birdee, Sujata G. Ayala, Kenneth A. Wallston

**Affiliations:** 1 Department of Medicine, Vanderbilt University, Nashville, TN 37232-8300 USA; 2 School of Nursing, Vanderbilt University, Nashville, TN 37232-8300 USA

**Keywords:** Yoga, Health-related quality of life, Mind-body practices

## Abstract

**Background:**

Mind-body practices such as yoga have been studied for their generally positive effects on health-related quality of life (HRQOL). The association between how a person practices yoga and the person’s HRQOL is not known.

**Materials and methods:**

Yoga practitioners were sent invitations to participate in an online survey via email. Yoga characteristics, HRQOL, and other sociodemographics were collected. Analyses of data from 309 consenting responders evaluated associations between yoga practice characteristics (use of yoga tools, length of practice, location, method, etc.) and the 10-item PROMIS Global Health scale for both physical and mental health components.

**Results:**

Multivariable regression models demonstrated higher mental health scores were associated with regular meditation practice, higher income, and the method of practicing in a community group class (versus one-on-one). Higher physical health scores were associated with length of lifetime practice, teacher status, Krishnamacharya yoga style, and practicing in a yoga school/studio (versus at home).

**Conclusions:**

Meditation practice in yoga is positively associated with mental health. Length of lifetime yoga practice was significantly associated with better physical health, suggesting yoga has a potential cumulative benefit over time. Different locations and methods of practice may be associated with varying effects on health outcomes. Comparative cross-sectional and longitudinal studies on the variations in yoga practice are needed to further characterize health benefits of yoga.

## Background

Mind-body practices are physical and mental exercises that are often used for health purposes [1]. Yoga, a mind-body practice originally derived from India, is one of the world’s most popular practices. In the United States, one out five adults practice yoga for health [1]. Individuals often report practicing yoga for general well-being [2]. Yoga uses three main tools for practice: movement, breathing, and meditation [3]. Practitioners use these tools to varying degrees. Many studies have shown improvements in health-related quality of life (HRQOL) from yoga practice including gains in both physical and mental health [4]. What is not clear is if characteristics of yoga practice, such as techniques used, yoga style, practice frequency, or level of experience, are associated with HRQOL. In the context of validating a new instrument to assess yoga self-efficacy, we collected additional data regarding how yoga was practiced by the participants, along with a measure of HRQOL [2]. The purpose of the present study was to perform a secondary analysis of that dataset to examine the associations between characteristics of yoga practice and HRQOL. We hypothesized that higher frequency of weekly yoga practice, length of practice, older age, and being a yoga teacher would be associated with higher physical and mental quality of life measures. Lastly, we expected no association between yoga style, location of practice, or method of practice with HRQOL.

## Methods

Data Source: We analyzed data collected during the development of the Yoga Self-Efficacy Scale which has been described in detail previously [5]. Yoga is represented by many different styles and traditions. Although our new measure was originally designed based on the competencies of a yoga practitioner according to the Krishnamacharya tradition (or Viniyoga), our sample included both practitioners of Viniyoga yoga and other yoga styles. The other types of yoga that were queried in the previous study include: Iyengar, Ashtanga, Bikram, Power, Kundalini, Sivananda, Kripalu, Anusara, and Hatha, and other. We dichotomized yoga styles into two groups: Viniyoga or other yoga styles. Yoga practitioners were recruited using national yoga association networks and the principal investigator’s personal contacts by email. Those who consented to participate were sent surveys through Research Electronic Data Capture (REDCap), a web application created at Vanderbilt for building and managing online surveys and databases for its use in research studies.

### The study sample’s characteristics have been previously published [5] and are summarized in Table 1. We selected the following measures for secondary analysis

**Table 1:** Sample characteristics (*n = 309)* ^*^

Characteristic	
Age (mean, S.D.)	51 (13)
Female Sex (n, %)	253 (82)
Race (n, %)	
White	262 (85)
Black	7 (2)
Asian	15 (5)
Some other race or missing	25 (8)
Hispanic, Latino or Spanish in origin (n, %)	15 (5)
Annual income (U.S. dollars) (n, %)	
Less than $24,999	34 (12)
$25,000-$44,999	50 (16)
$50,000-$74,999	48 (16)
$75,000-$99,999	46 (15)
$100,000 or more	78 (25)
Decline to answer	35 (11)
Education (n, %)	
High school graduate/GED/ or less	6 (2)
Vocational/technical school	8 (3)
Associate degree/some college	31 (10)
Bachelor’s degree	88 (29)
Advanced degree	158 (51)
Most frequent yoga style practiced (n, %)	
Krishnamacharya or Viniyoga	159 (52)
Hatha	53 (17)
Anusara	8 (3)
Ashtanga	9 (3)
Kripalu	4 (1)
Bikram	1 (<1)
Kundalini	1 (<1)
Other	31 (10)
Certified yoga teacher (n, %)	174 (56)
On average, how many times a week do you practice yoga? (mean, S.D.)	5 (2)
When you practice yoga, on average how many minutes? (mean, S.D.)	54 (20)
How long have you been practicing yoga? (n, %)	
More than 3 years	246 (80)
3 years or less	53 (17)
Health Related Quality of Life Sub-scores (mean, SD)	
Global Mental Health	15.01 (2.75)
Global Physical Health	16.51 (2.23)

#### Health-related quality of life

The 10-item PROMIS Global Health scale was administered to assess the physical and mental components of health related quality of life [6]. This scale can be scored into Global Mental Health (GMH) and Global Physical Health (GPH) subscales. A sample question from the GMH subscale is, “In general, how would you rate your mental health, including your mood and your ability to think?”[Likert scale: 5 = excellent to 1 = poor]. In the previous study, Cronbach’s alpha was 0.831 for GMH scores. An example from the GPH subscale is, “In general, please rate how well you carry out your everyday physical activities such as walking, climbing stairs, carrying groceries, or moving a chair [Likert scale: 5 = completely to 1 = not at all]. In the previous study, Cronbach’s alpha was 0.729 for GPH scores.

### Yoga practice characteristics and sociodemographics

A series of questions about characteristics of yoga practice were asked in a series of questions, including information about adherence, length of practice, and the perceived importance and practice of the three tools of yoga (breathing, movement, and meditation). Items concerning the three tools of yoga were ranked on a 9-point Likert scale ranging from 1 =”strongly disagree” to 9 = “strongly agree”. Perceived importance of each tool was assessed by asking if the tool was an important part of the respondent’s regular yoga practice (e.g., “Meditation is an important part of my regular yoga practice”), while practice of each tool was asked in the following fashion, for example, “My regular yoga practice includes meditation”. Participants were also queried on the duration of their yoga practice [less than 1 month, 1–3 months, 4–11 months, 1–3 years, more than 3 years]; and how many days a week they practiced [1–7 days]. Participants also self-reported their age, sex, yoga style, and if they were a certified yoga teacher.

### Analyses

Analyses were conducted using IBM SPSS Statistics Version 23. Descriptive statistics were used to assess perceived importance and practice of the three yoga tools (movement, breathing, and meditation), practice frequency, and length of practice. Pearson Chi Square analyses were conducted with sociodemographic variables and quality of life measures (GPH and GMH). Correlations between the practice of the three yoga tools and quality of life measures were calculated using Spearman’s correlation. We constructed multivariable linear regression models with the practice of the three yoga tools, certified yoga teacher status, length of practice, practice frequency, location of practice method of practice, method of practice, and yoga style as the independent variables affecting the physical and mental components of HRQOL. Age, gender, education level, income, and race were included as co-variates in all models.

## Results

### Sample characteristics by global PROMIS subscale scores

In Table 2, we report average GPH and GMH scores by the sociodemographic variables listed. GPH scores were significantly different by teacher status (certified teacher vs. not certified), with certified teachers scoring higher than non-certified practitioners. There was no significant difference between the two groups on GMH scores.

**Table 2:** Global PROMIS HRQOL subscale scores by selective demographic and yoga practice characteristics

	PROMIS Scores^a^ *N* = 291	
	Global Physical	Global Mental
Age		
25-35 (*n* = 49)	16.34 SD = 2.35	14.12 SD = 3.30
36-45 (*n* = 57)	16.21 SD = 2.22	14.21 SD = 2.34
46-55 (*n* = 61)	16.52 SD = 2.63	14.97 SD = 2.74
56-65 (*n* = 84)	16.93 SD = 1.97	15.56 SD = 2.51
66-75 (*n* = 36)	16.19 SD = 2.41	16.17 SD = 2.42
> 75 (*n* = 4)	16.63 SD = 3.90	15.75 SD = 3.40
Gender		
Male (*n* = 11)	16.74 SD = 2.16	14.74 SD = 3.16
Female (*n* = 253)	16.48 SD = 2.24	15.05 SD = 2.69
Race		
White (*n* = 258)	16.54 SD = 2.26	15.04 SD = 2.74
Other (*n* = 33)	16.30 SD = 2.05	14.70 SD = 2.89
Yoga Style		
Viniyoga Yoga (*n* = 158)	16.80 SD = 2.03	15.27 SD = 2.55
Other Yoga (*n* = 133)	16.17 SD = 2.41	14.69 SD = 2.95
Teacher Status		
Certified Teacher (*n* = 170)	16.89 SD = 2.08	15.05 SD = 2.50
Not Certified Teacher (*n* = 121)	15.98 SD = 2.33	14.95 D = 3.08

^a^Mann-Whitney U tests were calculated for all sociodemographics, Teacher status vs. GPH (U = 7516.5, *p* < 0.01) was the only significant relationship

### Correlations of yoga tools and global PROMIS subscale scores

Table 3 shows the correlations between the degree of practice of the three yoga tools (meditation, movement, and breathing) and GMH and GPH scores). GMH had significant correlations with both breathing and meditation whereas GPH only had a significant correlation with breathing.

**Table 3:** Spearman correlations of yoga tools with global PROMIS HRQOL subscale scores

HRQOL Subscale scores *N*=291		
Tools	Global Mental	Global Physical
Regular Yoga Practice Includes Meditation	.16^**^	.09
Regular Yoga Practice Includes Movement	.09	.02
Regular Yoga Practice Includes Breathing Technique	.20^**^	.14^*^

*p < 0.05 **p <0.01

### Regression models predicting global PROMIS subscale scores

Table 4 reports the regression model we built to understand the relationship between GMH and selected independent variables. The regression model considers characteristics of yoga practice and sociodemographic factors as independent variables with GMH serving as the dependent variable. Significant predictors were: age, regular practice of the meditation, and method of practicing in a community group class (versus one-on-one). The model was found to explain a significant amount of variance in GMH scores (*R* ^*2*^ *= 0.15, R* ^*2*^_*adjusted*_ *= 0.13; F (7,248) = 6.21, p <0.05)*.

**Table 4:** Global mental HRQOL subscale score correlations and regression model

	Correlations	Regression model		
Variable	*r* ^a^	B	SE B	β
Age	0.29**	0.056	0.013	0.265**
Race	0.04	0.201	0.541	0.022
Gender	0.04	0.293	0.510	0.034
Income	0.21**	0.125	0.098	0.083
Education	0.09	0.618	0.472	0.080
Regular practice includes meditation (yoga tool)	0.18**	0.231	0.087	0.159**
Method of Practice- community group class	0.12*	0.797	0.376	0.128*
*R*			0.386	
*R* ^2^			0.149	
*R* ^*2*^_*adjusted*_			0.125	
ANOVA *F(7,248)*			6.211**	

^a^Correlation coefficient

*p <0.05 **p < 0.01

The parallel regression model for GPH as the dependent variable is shown in Table 5. Significant independent variables included length of practice, teacher status, yoga style, and practicing in a yoga school/studio (versus at home) were significantly associated with GPH. This model was also found to explain a significant amount of variance in GPH scores (*R* ^*2*^ *= 0.14, R* ^*2*^_*adjusted*_ *= 0.11; F (9, 246) = 4.39, p <0.05)*.

**Table 5:** Global physical HRQOL subscale score correlations and regression model

	Correlations	Regression model		
Variable	*r* ^a^	B	SE B	β
Age	0.07	0.002	0.011	0.012
Race	0.04	0.171	0.442	0.023
Gender	0.03	-0.518	0.420	-0.074
Income	0.07	0.081	0.080	0.066
Education	0.11	0.480	0.385	0.077
Length of Yoga Practice	0.22**	0.576	0.214	0.183*
Teacher Status	0.20**	0.832	0.334	0.180*
Viniyoga	0.14**	0.694	0.283	0.153*
Location of Practice- yoga studio/school	0.07	0.910	0.331	0.178**
*R*		0.372		
*R* ^*2*^		0.138		
*R* ^*2*^_*adjusted*_		0.107		
ANOVA *F(9,246)*		4.394**		

^a^Correlation coefficient

*p <0.05 **p < 0.01

## Discussion

Our analysis demonstrated that specific characteristics of yoga practice are associated with different components of HRQOL. Global mental health scores were associated with regular practice of meditation and community group instruction (versus one-on-one instruction), while global physical health scores were associated with longer length of yoga practice, Viniyoga style, being a certified yoga teacher, and practicing in a yoga studio/school. Of the three yoga tools (meditation, movement, and breathing), GMH had a positive correlation with breathing and meditation, whereas GPH had a positive correlation with breathing only. These results suggest that variations in yoga practice are associated with different physical and mental health statuses.

The specific effect of yoga on mental health has been a popular topic of research, with previous studies showing a positive relationship between the two [4, 7]. Yoga interventions have been shown to reduce stress, and yoga practitioners tend to demonstrate a higher level of mindfulness than non-practitioners [8–10]. Complimentary to these findings, our analyses demonstrated that participants who reported regular use of meditation or breathing in their yoga practice tended to have higher mental health scores. In the regression model predicting mental HRQOL, regular use of meditation was the only yoga tool to have a significant unique relationship with mental health status, albeit a small one. Because meditation has been demonstrated to improve mental health and is a common component of yoga practice, further research is needed to identify if meditation is the major causal mechanism for improving mental health status among yoga practitioners. The model predicting physical HRQOL scores was found to be positively associated with the length of yoga practice. We defined length of yoga practice as the total time a practitioner has been practicing yoga, whereas frequency of practice was defined as how often he/she practices in an average week. Previous research shows conflicting results. One study demonstrated that frequency of weekly yoga practice was better at defining health outcomes than length of yoga practice, whereas another study determined that frequency of practice (one versus two practice days) had little difference in health outcomes [11, 12]. In our analyses, length of practice had the highest correlation with physical HRQOL status among the variables tested. One possible hypothesis for this observation may be that many of our participants were yoga teachers who had been practicing for many years benefiting from a cumulative effect on overall physical HRQOL over time.

Other findings to note include the significance of the location of practice. Practicing in a yoga school/class setting was positively associated with physical HRQOL. There may be multiple reasons for this association. Individuals who practice at a yoga school may benefit from group effects where they are socially motivated to participate, resulting in higher adherence and intensity. Secondly, attending class at a school may provide access to other physical exercise programs offered. Lastly, yoga classes in schools may emphasize physical fitness more than home or other places of practice. Further research is necessary to examine if classes in yoga schools are optimal for improving physical well-being. Method of practice was found to be important for mental HRQOL. Specifically, those who reported practicing yoga in community group classes had higher GMH scores. This requires further examination, as those individuals who seek community group instruction may especially benefit from group settings. Both of these findings suggest that location and method of practice may influence the HRQOL status of an individual. To our knowledge this has not been previously reported in the literature.

### Limitations

Our study has several limitations. The survey responders consisted of a fairly homogenous group (predominantly white, female, and educated) [8]. Although these demographics are consistent with the general yoga practicing population in the United States, we have not assessed the association of these characteristics (white, female, and educated) with increased regular practice of yoga, which may factor into the higher GPH/GMH scores demonstrated in the study [13]. In addition, the method employed for recruiting survey responders may have led to selection bias as it was focused on a specific style of yoga (Krishnamacharya or Viniyoga), and most responders were experienced yoga teachers. Many of the relationships we found, although statistically significant, may not be considered as strong, thus limiting our interpretations of our results. Again, this may be attributed to the homogeneity of our study sample and warrants further investigation of these relationships. Thus, these findings may not generalize to different styles of yoga or to less experienced practitioners. Lastly, these data are cross-sectional, thus we cannot reliably infer any causal relationships from our secondary analyses.

## Conclusion

The practice of yoga for recreation and wellness is very popular in the United States. Understanding the effects of specific features of yoga practice on physical and mental health quality of life may enable teachers to ask more pertinent questions in order to better understand its observed health outcomes. Similarly, teachers may offer counsel on mind-body therapies or tailor yoga practices based on the specific needs of the student. If more data regarding the differential effects of yoga was known, yoga teachers may be able to provide more direction on frequency, intensity, and type, similar to the guidance clinicians provide with other types of exercise. For example, regular use of meditation in yoga practice may have a positive effect on mental HRQOL, whereas practicing at a yoga school/studio may have a positive effect on physical HRQOL. More research is required to better understand the relationship between techniques, length of practice, and setting of yoga practice and the potential differential effects on quality of life in regards to physical and mental health.

## Abbreviations

GMH: Global mental health
GPH: Global physical health
HRQOL: Health-related quality of life
